# Supplementary material for: Perturbations of the Proteome and of Secreted Metabolites in Primary Astrocytes from the hSOD1(G93A) ALS Mouse Model
Source: Int J Mol Sci. 2021 Jun 29;22(13):7028. doi: 10.3390/ijms22137028 (PMC8268687; doi:10.3390/ijms22137028)
Supplement: Supplementary file 1 [file ijms-22-07028-s001.zip › Supplementary Figures_IJMS_rev1.pdf]

Altered proteome and secreted metabolome in primary astrocytes from the hSOD1(G93A) ALS mouse model

Roberto Stella[1], Raphael Severino Bonadio[2], Stefano Cagnin[2, 3], Maria Lina Massimino[4], Alessandro Bertoli[4,5,6,*], Caterina Peggion[6,*]

Supplementary Materials

Supplementary Figures

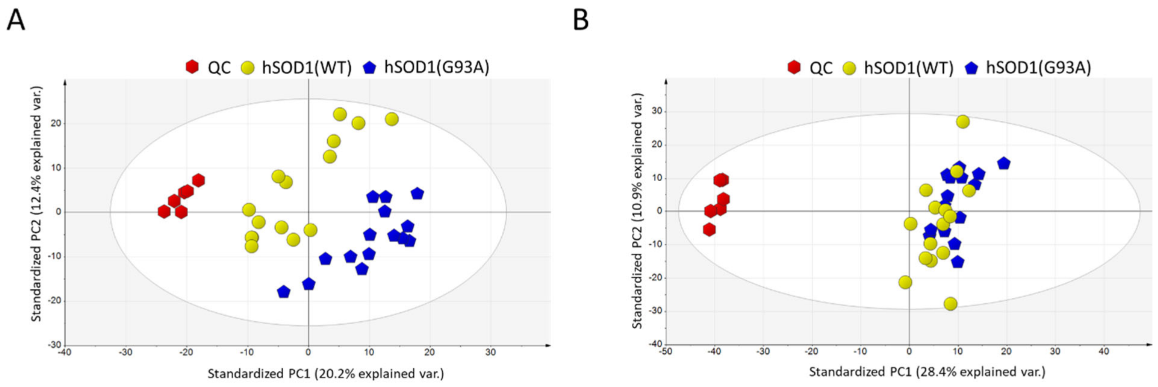

Supplementary Figure S1 – Score plots of metabolomics data. Principal component analysis (PCA) score plots of the metabolome data of CMs of hSOD1(WT) (*yellow circles*), hSOD1(G93A) (*blue pentagons*) primary astrocytes and of QC samples (*red hexagons*) acquired in the negative (panel A) and positive (panel B) ionization condition. Other details are as in Figure 1.

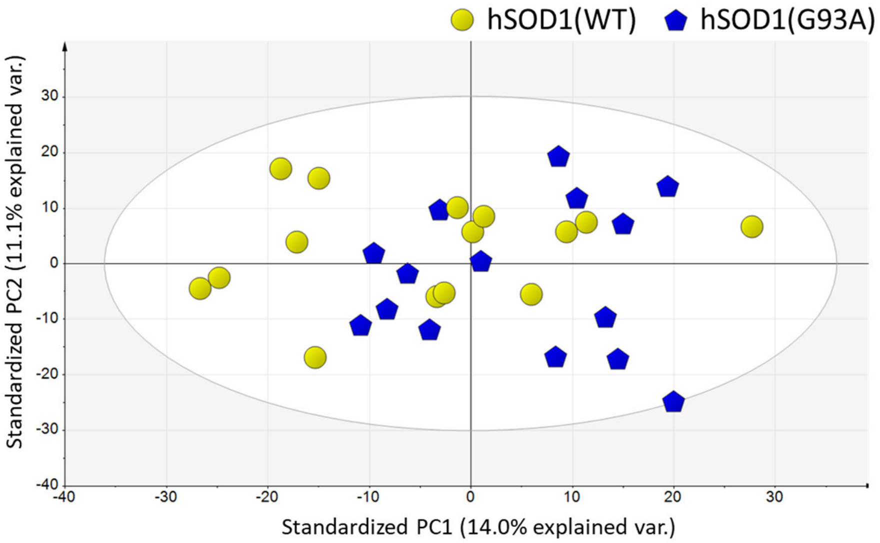

Supplementary Figure S2 - Score plot of metabolomics data acquired in the positive ionization mode. PCA score plot of metabolomics data of CMs of hSOD1(WT) (*yellow circles*), hSOD1(G93A) (*blue pentagons*) primary astrocytes acquired in the positive ionization condition. Other details are as in Figure 1

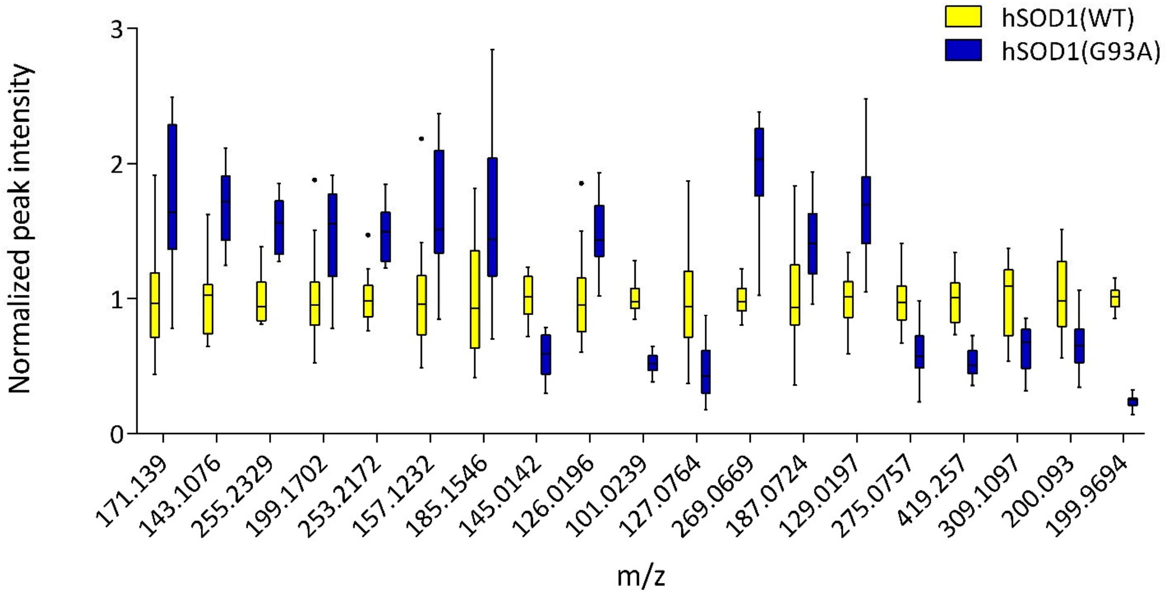

Supplementary Figure S3 – Altered amounts of 19 metabolites in the CM of hSOD1(G93A) astrocytes. Box-and-whisker plot showing the normalized abundance of the 19 significantly affected metabolites in the CM derived from hSOD1(WT) (*yellow box*) and hSOD(G93A) (*blue box*) astrocytes. For each metabolite, the corresponding measured m/z is reported in the graph (see Supplementary Table 1, in which for each m/z the corresponding systematic name is reported). The bottom and top of the boxes represent the 5th and 95th percentiles, the line in the middle indicates the median. n=15 for each hSOD1 genotype. Statistical significance was calculated with the Wilcoxon rank test and p-value of each metabolite is reported in Supplementary Table 1.

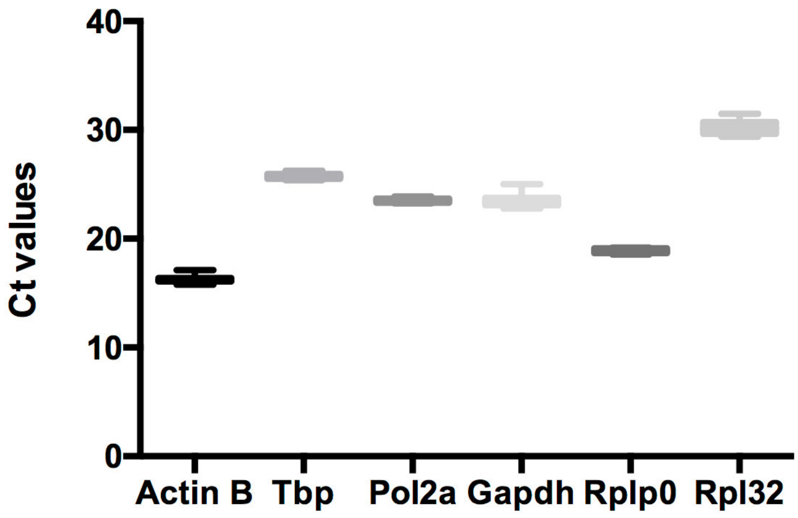

Supplementary Figure S4 – Identification and evaluation of reference genes for qRT-PCR analysis in primary spinal astrocytes. Analysis of cycle threshold (Ct) values of the 6-candidate reference genes across all samples. Expression data displayed as Ct values for each reference gene in all astrocyte samples. Data are reported as mean ± standard deviation.
