# Supplementary material for: Perturbations of the Proteome and of Secreted Metabolites in Primary Astrocytes from the hSOD1(G93A) ALS Mouse Model
Source: Int J Mol Sci. 2021 Jun 29;22(13):7028. doi: 10.3390/ijms22137028 (PMC8268687; doi:10.3390/ijms22137028)
Supplement: Supplementary file 1 [file ijms-22-07028-s001.zip › supplementary tables S1 S2 S3 IJMS Rev1.pdf]

Name (Manually annotated)	KEGG pathway/*compounds*	Formula	G93A/WT ratio	p-value	RT (min)	*m/z*	Adduct
Decanoic acid	Fatty acid biosynthesis	$C_{10}H_{20}O_2$	1.703	2.85E-04	1.11	171.139	[M-H]-
Octanoic acid		$C_8H_{16}O_2$	1.672	1.43E-07	1.112	143.1076	[M-H]-
Hexadecanoic acid		$C_{16}H_{32}O_2$	1.663	1.73E-08	1.082	255.2329	[M-H]-
Dodecanoic acid		$C_{12}H_{24}O_2$	1.632	2.00E-03	1.101	199.1702	[M-H]-
Hexadecenoic acid		$C_{16}H_{30}O_2$	1.521	2.08E-07	1.084	253.2172	[M-H]-
Nonanoic acid	*Fatty acids*	$C_9H_{18}O_2$	1.572	4.11E-04	1.115	157.1232	[M-H]-
Undecanoic acid	*Fatty acids*	$C_{11}H_{22}O_2$	1.549	3.99E-03	1.105	185.1546	[M-H]-
Oxoglutaric acid	Alanine, aspartate and glutamate metabolism	$C_5H_6O_5$	0.58	3.38E-08	1.41	145.0142	[M-H]-
Oxoglutaramic acid		$C_5H_7NO_4$	1.5	9.66E-05	8.083	126.0196	[M-H2O-H]-
Succinic acid semialdehyde		$C_4H_6O_3$	0.529	2.92E-13	1.42	101.0239	[M-H]-
Isoprene	Biosynthesis of terpenoids and steroids	C_5H_8	0.452	5.72E-04	1.352	127.0764	[M+CH3COO]-
D-Lombricine	Glycine, serine and threonine metabolism	$C_6H_{15}N_4O_6P$	2.081	1.09E-08	6.656	269.0669	[M-H]-
Ectoine		$C_6H_{10}N_2O_2$	1.508	1.89E-03	8.084	187.0724	[M+FA-H]-
Citraconic acid	Valine, leucine and isoleucine biosynthesis	$C_5H_6O_4$	1.667	4.00E-06	1.401	129.0197	[M-H]-
Galactosylglycerol	Glycerolipid metabolism	$C_9H_{18}O_8$	0.586	5.11E-06	1.338	275.0757	[M+Na-2H]-
Phosphatidic acid		$C_{24}H_{34}N_6O_2$	0.502	8.47E-08	1.269	419.257	[M-H2O-H]-
Gamma-L-glutamyl-L-tyrosine	Glutathione metabolism	$C_{14}H_{18}N_2O_6$	0.615	2.29E-04	9.16	309.1097	[M-H]-
Pantothenic acid	Pantothenate and CoA biosynthesis	$C_9H_{17}NO_5$	0.663	7.48E-04	1.274	200.093	[M-H2O-H]-
Cysteine-S-sulfate	Cysteine and methionine metabolism	$C_3H_7NO_5S_2$	0.245	2.18E-20	8.079	199.9694	[M-H]-

Supplementary Table S1 – Altered metabolites in CM of hSOD1(G93A) primary astrocytes. Annotation and KEGG metabolic pathways of CM metabolites detected in the ESI – polarity whose expression ratio (hSOD1(G93A) vs hSOD1(WT) astrocytes) was < 0.66 and > 1.50 (p-value < 0.05, Wilcoxon rank-sum test) were reported. The measured *m/z*, the chromatographic retention time (RT), and proposed adduct were detailed.

Uniprot Accession	Gene Name	Protein Name	ratio G93A/WT	p-value
**P10605**	**Ctsb**	**Cathepsin B**	**1.307**	**0.006; 0.049**
**Q9WUU7**	**Ctsz**	**Cathepsin Z**	**1.484**	**0.008; 0.049**
O70370	Ctss	Cathepsin S	1.374	0.014; **0.049**
P99026	Psmb4	Proteasome subunit beta type-4	1.321	0.005; **0.049**
**Q9CPS5**	**Psmd8**	**26S proteasome non-ATPase regulatory subunit 8**	**1.356**	**0.036; 0.049**
A0A0R4J0I9	Lrp1	Low density lipoprotein receptor-related protein 1	1.987	0.043; **0.049**
O89053	Coro1a	Coronin-1A	1.329	0.001; **0.049**
**P13020**	**Gsn**	**Gelsolin**	**1.344**	**0.012; 0.049**
O70200	Aif1	Allograft inflammatory factor 1	1.356	0.036; **0.049**
Q5SW88	Rab1a	RAB1A, member RAS oncogene family	1.467	0.010; **0.049**
P20060	Hexb	Beta-hexaminidase subunit beta	1.429	0.005; **0.049**
A0A087WS56	Fn1	Fibronectin	1.612	0.008; **0.049**
Q9DAK9	Phpt1	14 kDa phophohistidine phophatase	1.388	0.020; **0.049**
Q64282	Ifit1	Interferon-induced protein with tetratricopeptide repeats 1	1.590	0.032; **0.049**
Q9D115	Znf706	Zinc finger protein 706	2.115	0.047; **0.049**
P19324	Serpinh1	Serpin H1	1.321	0.005; **0.049**
Q8C845	Efhd2	EF-hand domain-containing protein D2	1.514	0.024; **0.049**
Q91VW3	Sh3bgrl3	SH3 domain-binding glutamic acid-rich-like protein 3	1.384	0.041; **0.049**

Supplementary Table S2- Up-regulated proteins in hSOD1(G93A) primary spinal astrocytes. Among the 640 proteins identified and quantified in hSOD1(WT) and hSOD1(G93A) astrocytes, the only proteins whose expression ratio (G93A vs WT cells) was >1.33 (p-value <0.05, two-tailed t-test) are reported. UNIPROT accessions, gene names, and protein names are reported in the first three columns. In the last column the p-values obtained with either a two-tailed Student's t-test (in black) and the Wilcoxon Rank sum test (un-paired, two-sided; confidence level 0.95) (in blue) are reported. Subsequent validation analysis by Western blot was performed only for proteins reported in bold.

Uniprot accession	Gene name	Protein Name	Ratio G93A/WT	p-value
**O09131**	**Gsto1**	**Glutathione S-transferase omega-1**	**0.420**	**0.033; 0.049**
Q9CPU0	Glo1	Lactoylglutathione lyase	0.648	0.046; **0.049**
**P97494**	**Gclc**	**Glutamate--cysteine ligase catalytic subunit**	**0.672**	**0.043; 0.049**
O88531	Ppt1	Palmitoyl-protein thioesterase 1	0.646	0.022; **0.049**
Q8VCQ8	Cald1	Caldesmon 1	0.743	0.001; **0.049**
Q3TT92	Dpysl3	Dihydropyrimidinase-related protein 3	0.751	0.031; **0.049**
P97315	Csrp1	Cysteine and glycine-rich protein 1	0.764	0.011; **0.049**
G3UVV4	Hk1	Hexokinase 1, isoform CRA_f	0.681	0.032; **0.049**
Q08091	Cnn1	Calponin-1	0.701	0.001; **0.049**
Q8BZF8	Pgm5	Posphoglucomutase-like protein 5	0.706	0.049; **0.049**
D3YYE1	Anp32a	Acidic leucine-rich nuclear phphoprotein 32 family member A (Fragment)	0.710	0.010; **0.049**
Q9R0P9	Uchl1	Ubiquitin carboxyl-terminal hydrolase isozyme L1	0.710	0.040; **0.049**
G3X9T8	Cp	Ceruloplasmin	0.733	0.047; **0.049**
P37804	Tagln	Transgelin	0.739	0.021; **0.049**
A2AIM4	Tpm2	Tropomyin beta chain	0.759	0.005; **0.049**
P50543	S100a11	Protein S100-A11	0.770	0.030; **0.049**
O55022	Pgrmc1	Membrane-associated progesterone receptor component 1	0.624	0.018; **0.049**
P51655	Gpc4	Glypican-4	0.667	0.004; **0.049**
E9PWK1	Ephx1	Epoxide hydrolase	0.749	0.045; **0.049**
H3BLJ9	Esd	S-formylglutathione hydrolase	0.621	0.046; **0.049**

Supplementary Table S3 - Down-regulated proteins in hSOD1(G93A) primary astrocytes. Among the 640 proteins identified and quantified in hSOD1(WT) and hSOD1(G93A) astrocytes, the only proteins whose expression ratio (G93A vs WT cells) was <0.77 (p-value <0.05, two-tailed t-test) are reported. Other details are as in Supplementary Table 2.
